# Growth model and structure evolution of Ag layers deposited on Ge films

**DOI:** 10.3762/bjnano.9.9

**Published:** 2018-01-08

**Authors:** Arkadiusz Ciesielski, Lukasz Skowronski, Ewa Górecka, Jakub Kierdaszuk, Tomasz Szoplik

**Affiliations:** 1 University of Warsaw, Faculty of Physics, Pasteura 5 str., 02-093 Warsaw, Poland; 2 UTP University of Science and Technology, Institute of Mathematics and Physics, Kaliskiego 7 Str. 85-796 Bydgoszcz, Poland; 3 University of Warsaw, Department of Chemistry, Pasteura 1 str., 02-093 Warsaw, Poland

**Keywords:** germanium, segregation, self-assembly, silver, thin films

## Abstract

We investigated the crystallinity and optical parameters of silver layers of 10–35 nm thickness as a function 2–10 nm thick Ge wetting films deposited on SiO_2_ substrates. X-ray reflectometry (XRR) and X-ray diffraction (XRD) measurements proved that segregation of germanium into the surface of the silver film is a result of the gradient growth of silver crystals. The free energy of Ge atoms is reduced by their migration from boundaries of larger grains at the Ag/SiO_2_ interface to boundaries of smaller grains near the Ag surface. Annealing at different temperatures and various durations allowed for a controlled distribution of crystal dimensions, thus influencing the segregation rate. Furthermore, using ellipsometric and optical transmission measurements we determined the time-dependent evolution of the film structure. If stored under ambient conditions for the first week after deposition, the changes in the transmission spectra are smaller than the measurement accuracy. Over the course of the following three weeks, the segregation-induced effects result in considerably modified transmission spectra. Two months after deposition, the slope of the silver layer density profile derived from the XRR spectra was found to be inverted due to the completed segregation process, and the optical transmission spectra increased uniformly due to the roughened surfaces, corrosion of silver and ongoing recrystallization. The Raman spectra of the Ge wetted Ag films were measured immediately after deposition and ten days later and demonstrated that the Ge atoms at the Ag grain boundaries form clusters of a few atoms where the Ge–Ge bonds are still present.

## Introduction

Silver is a noble metal with lowest loss in the visible to the near-infrared wavelengths; therefore, the surface plasmon polariton (SPP) wave propagation length crucial for plasmonic devices is greatest at Ag/dielectric interfaces [1–3]. A pure Ag layer of 35 nm thickness has an imaginary part of permittivity lower than 1 within the 315–827 nm range [4]. Therefore, silver is widely used in plasmonic sensors [5–7], as substrates for surface enhanced Raman scattering (SERS) [8–9], as inclusion in solar cells [10–12] and in other plasmonic devices [13–14]. The SPP wave propagation length depends on the permittivity of the metal film, but also on its surface roughness, which is responsible for scattering losses. Thin silver layers deposited on glass substrates usually exhibit an island growth [15].

One way of fabricating smooth and thermally stable Ag-based layers of thickness less than 15 nm on fused silica substrates is magnetron cosputtering of silver and aluminum. Surface root-mean-square (RMS) roughness of 15 nm Al-doped Ag films with an Al atomic concentration of 4% have been recently reported to be equal to 0.4 nm [16]. However, in spite of scattering loss reduction, the measured imaginary part of permittivity of a 7 nm Al-doped Ag film is three-fold higher than that of a 30 nm pure Ag in the 400–1700 nm spectral range [17]. On the other hand, if silver films are thermally or electron beam (e-beam) evaporated under optimum conditions [18] and with a Ge nucleation film, they are smoother than layers of similar thickness deposited directly on glass [19] and have significantly lower mean grain size [20]. The reduction of scattering losses due to a temporary decrease of RMS roughness to 0.2 nm was reported for an e-beam evaporated 10 nm Ag layer on a sapphire substrate at room temperature with a 1 nm germanium wetting film [18]. Recently, it was observed that the germanium atoms, which form the nucleation film, efficiently segregate [21] through the silver structure towards the surface [22–23], which results in additional bands in the permittivity spectrum as well as an increase in specific resistivity [24]. Moreover, a hypothesis has been stated, that these two effects are connected by a cause-and-effect relationship [4]. Here we verify this hypothesis by performing XRD and XRR measurements to obtain the mean grain size and density profile of 35 nm thick Ag layers seeded with 2 nm thick Ge films with and without temperature treatment. To investigate the kinetics of the segregation process, we perform time-dependent transmission and ellipsometry measurements for Ag samples deposited on Ge films with thicknesses varying from 2 to 10 nm. This allows to determine the rate of Ge segregation into the Ag polycrystalline structure, but also to assess at which Ag-to-Ge ratio these samples can be treated as silver grains decorated with Ge atoms and when should they be treated as an elemental mixture. To verify whether Ge atoms present in the Ag grain boundaries are individual atoms or small clusters, Raman spectra of Ge wetted, 20 nm thick Ag layers were recorded immediately after deposition as well as 10 days later. To avoid corrosion, all samples were capped with a 3 nm thick LiF overlayer, except the ones measured with Raman microscopy, which have been kept at a pressure below 10^−3^ Torr until the measurement.

## Results and Discussion

### Crystalline structure of Ag layers grown on Ge wetted silica substrates

**Figure 1:**
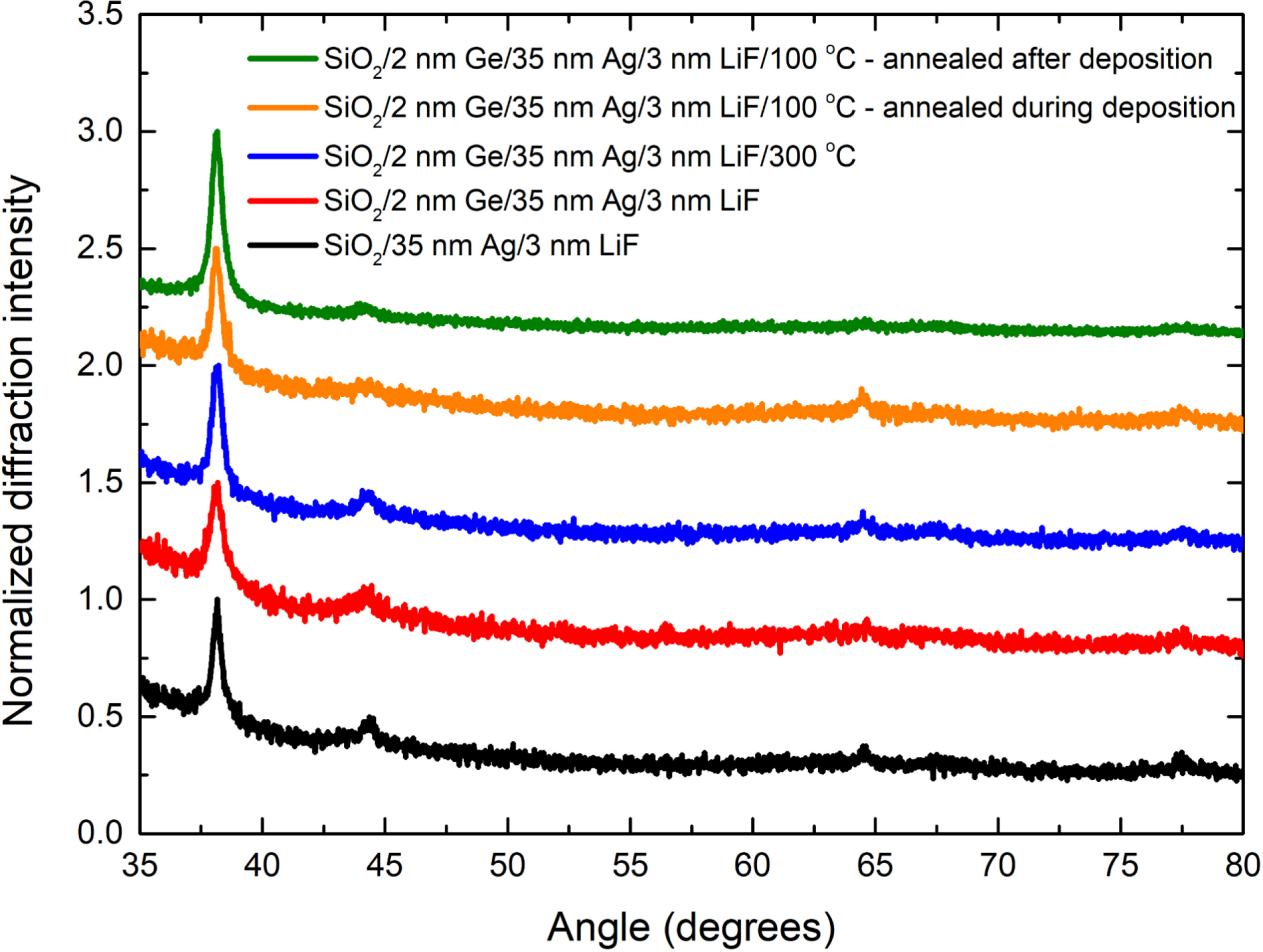
XRD spectra of the investigated silver films. The intensity of each spectrum was normalized to the maximum intensity at the (111) diffraction peak in that spectrum. Each curve is offset by 0.5 with respect to the previous one.

Figure 1 presents the XRD spectra of the investigated silver films. In each case, there is a strong diffraction peak at 38.2°, which corresponds to the (111) plane spacing. For a typical powder sample [25], the intensity of the (200) peak at 44.3° should be about half the intensity of the (111) peak. In our samples, the intensity of the (200) peak is much smaller, which was also observed by Logeeswaran et al. [20]. Furthermore, (220) and (311) peaks at 64.3° and 77.6°, respectively, are nearly nonexistent, therefore we assume that our samples are composed almost exclusively of grains which are oriented with a (111) plane with respect to the growth axis.

**Figure 2:**
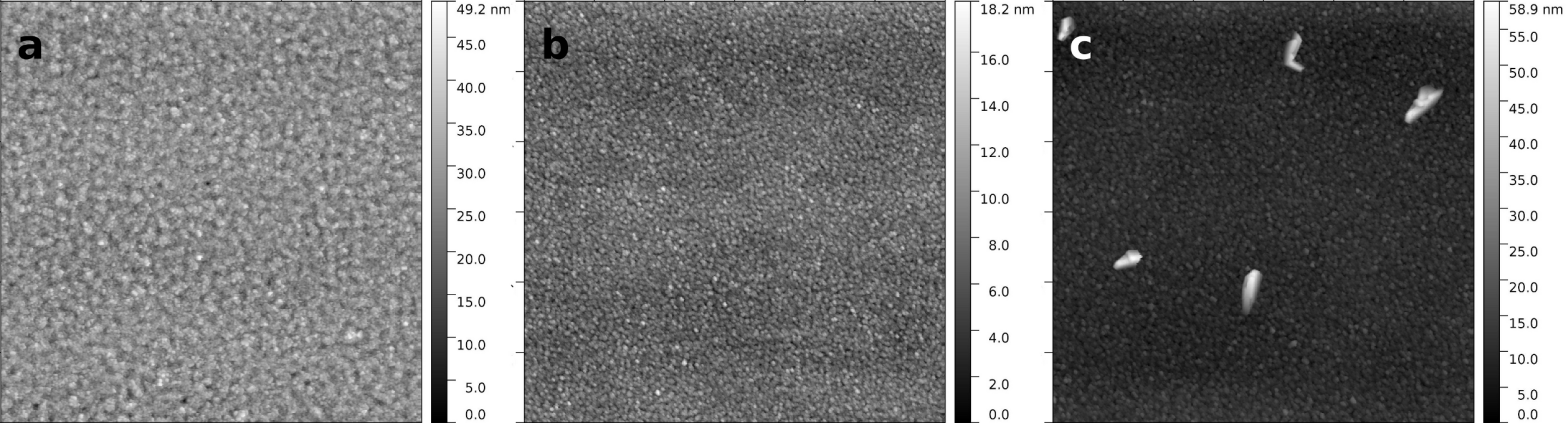
3 × 3 µm^2^ AFM scans of 35 nm thick silver films. (a) Non wetted film, (b) Ge wetted, not annealed film, (c) Ge wetted film annealed at 300 °C.

In such a case it is difficult to distinguish the contribution from the change in the grain size and the contribution from the microstrain in the silver grains. However, microstrain should significantly alter the intensities of both interband transition peaks in the silver permittivity spectra, which we do not observe. Furthermore, atomic force microscopy (AFM) scans show that the grain size indeed decreases when the Ag layer is deposited on a Ge wetting film (Figure 2b) with respect to the non wetted film (Figure 2a), which is in consistency with the previous findings [4,19–20,22,24].

Table 1 shows the AFM- and XRR-derived surface roughness root-mean-square (RMS) values as well as XRD-derived average grain size and lattice constant for several silver layers deposited on germanium wetting films. The RMS values derived from the AFM scans generally match well with the RMS values estimated from the XRR model – the deviations are less than 0.3 nm, with the exception of the sample annealed at 300 °C. Detailed information about this comparison can be found in the experimental section. The grain size of the 35 nm thick Ag layer on silica is as large as 18 nm which is half of its thickness. This is because the adhesion of silver to glasses is relatively low and cohesion of Ag adatoms dominates to induce Volmer–Weber growth. During the deposition process, clusters of silver adatoms behave as liquid droplets with high wetting angle. The measured height of these islands was 18.6 nm in the case of depositing an equivalent of 12 nm thick silver layer [15], and for thicker layers, the height of the nanocrystals may be even greater. In a recent work [4], we have modeled a 35 nm thick silver layer with a smooth surface to determine its optical parameters and we had a good agreement with the experimental data, therefore, we can assume that such a layer is fully continuous. This means that the 35 nm thick coating consists of 1–3 silver grain sublayers. Utilizing a 2 nm thick Ge interlayer considerably reduces the grain size and so, the 35 nm thick coating consists of 4–5 grain sublayers which naturally results in decreased roughness of the silver surface.

**Table 1:** XRD results and roughness of the samples.^a^

sample	grain size [nm]	lattice constant [Å]	RMS roughness derived from AFM scans/XRR spectra [nm]

SiO_2_/35 nm Ag/3 nm LiF	18	4.084	3.6/3.6
SiO_2_/2 nm Ge/35 nm Ag/3 nm LiF	9	4.087	1.4/1.3
SiO_2_/2 nm Ge/35 nm Ag/3 nm LiF/ 100 °C – annealed after deposition	16	4.086	2.2/2.0
SiO_2_/2 nm Ge/35 nm Ag/3 nm LiF/ 100 °C – annealed during deposition	17	4.085	2.2/2.0
SiO_2_/2 nm Ge/20 nm Ag/3 nm LiF/ 300 °C	18	4.084	2.4 (4.4)^b^/3.8

^a^XRD-determined average grain size and lattice constant as well as RMS roughness derived from AFM scans (before slash) and XRR spectra (after slash) for 35 nm thick silver layers grown on SiO_2_ substrates with 2 nm thick Ge wetting interlayer. ^b^The RMS = 4.4 nm is calculated from the AFM image taking into account the few large grains present in Figure 2c. For comparison, the values for the sample without Ge are provided.

**Figure 3:**
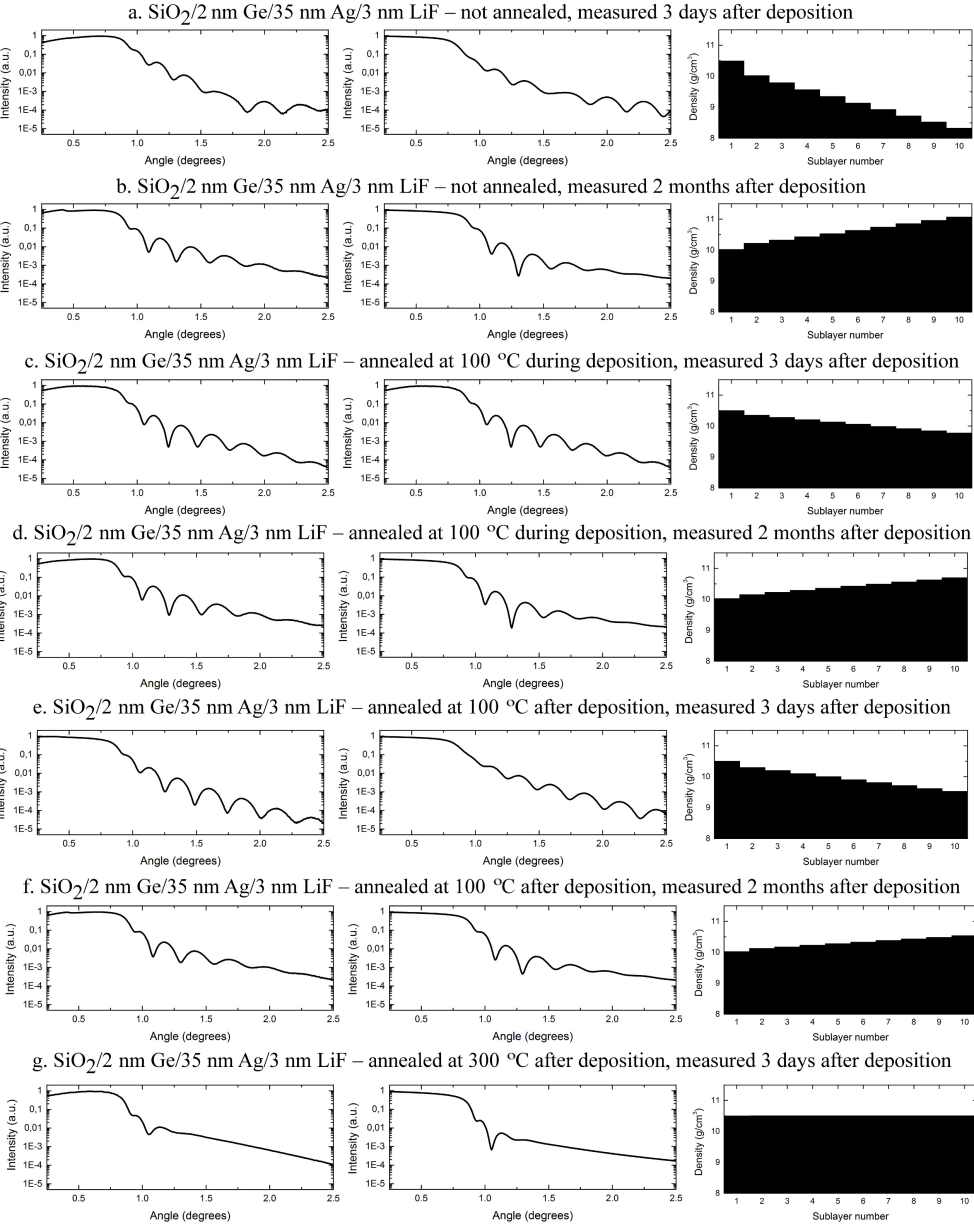
Measured XRR spectra (left column), fitted curves (middle column) as well as silver layer density profiles extracted from the modeled curves (right column) for differently processed Ag films deposited on silica substrates and Ge wetting layers. The silver volume in the model was divided into ten sublayers and the density slope was set to exponential. Sublayer 1 is the one at SiO_2_/Ag interface, while sublayer 10 is at Ag/LiF interface. It is worth noting that for all measurements performed 3 days after the deposition of the samples, it was necessary to consider an independent Ge interlayer between SiO_2_ substrate and silver, while for measurements performed after 2 months, it was not.

**Figure 4:**
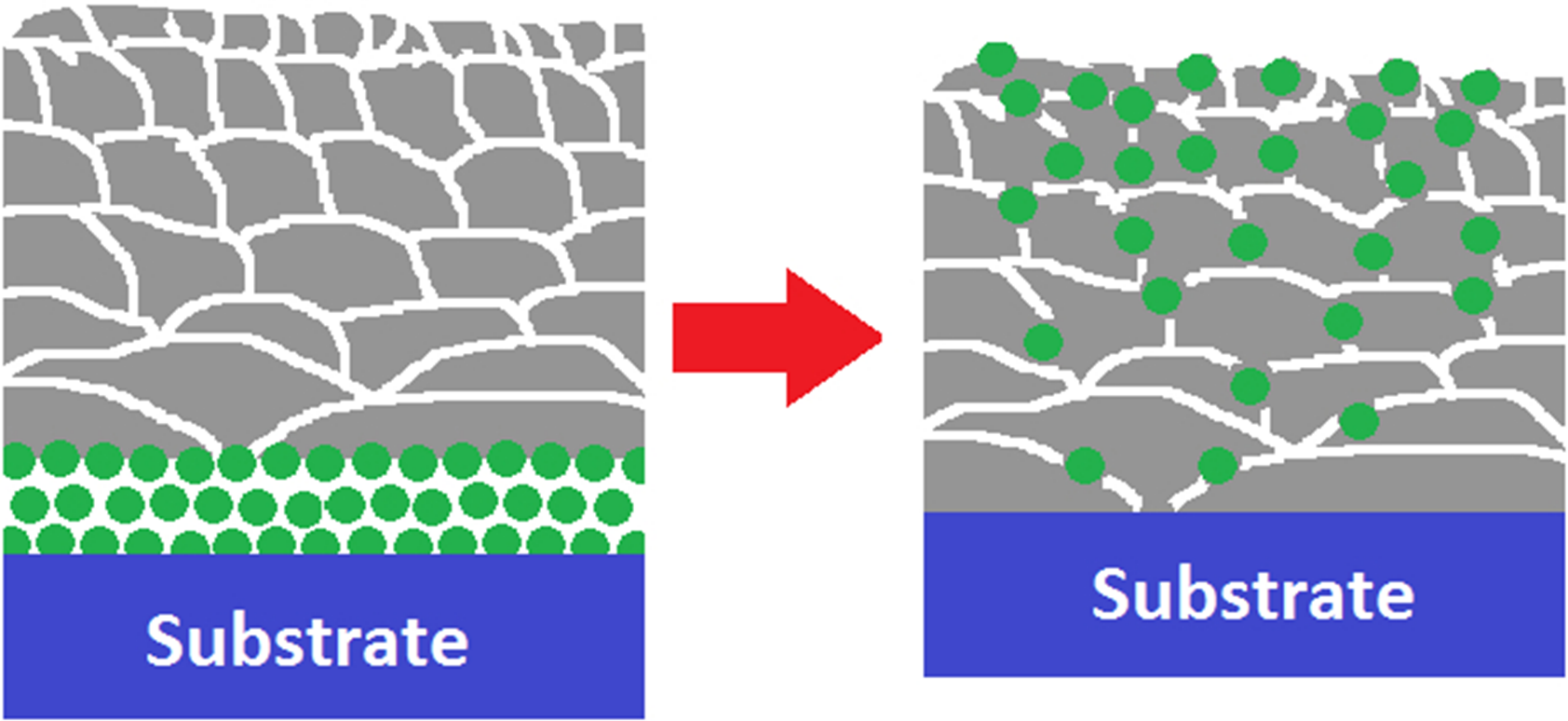
Scheme of the structure of the silver layers grown on Ge wetting films before (left) and after (right) the segregation process occurs. Large Ag grains (gray) seeded with Ge nanoclusters (green) are present at the SiO_2_/Ge/Ag interface, and their size decreases with increasing distance from the substrate.

With the increased number of grains, the surface of the grain boundaries and volume of voids also increases. As hypothesized in [4], Ge atoms located in the silver grain boundaries or in the voids have lower free enthalpy than Ge atoms at the SiO_2_/Ag interface. The increased number of grain boundaries is one of the reasons for the efficient segregation of germanium into the structure of the silver layers. However, the density profile of the silver layer is equally important. Figure 3a–g show the measured and modeled XRR spectra of differently processed, 35 nm thick silver layers as well as their density profiles extracted from the models. In each case, the best fit was acquired by dividing the silver into 10 sublayers and assuming the exponential change in the layer density. In the case of the non-annealed, 35 nm thick Ag layer deposited on Ge and measured 3 days after deposition (Figure 3a) the density decreases from 10.5 g/cm^3^ (which is the density of a bulk silver) at the SiO_2_/Ag interface to 8.5 g/cm^3^ at the Ag/LiF interface. This indicates a high non-uniformity in the sizes and shapes of the grains and the varying number of voids. Since germanium serves as a nucleation film for silver, the clusters of silver atoms behave as liquid droplets with low wetting angle as opposed to the case without using Ge [26–27]. Therefore, the silver grains located directly over the SiO_2_/Ge/Ag interface are large and flat – the in-plane (horizontal) sizes are large, but the out-of-plane (vertical) size is small. With the large in-plane grain sizes, there are few voids at the grain boundaries and so, the density of the layer is very close to the density of the bulk silver. During deposition, with increasing distance from the SiO_2_/Ge/Ag interface, the interaction of Ag and Ge atoms weakens and the cohesion force between Ag atoms starts to dominate. This leads to the formation of more numerous, smaller and compact grains. With more grains, the number of voids at grain boundaries also increases, which results in the decrease of the silver layer density (Figure 3a,c,e). The resulting structure of the silver layer grown on Ge wetting film is illustrated in Figure 4. Large and flat nanocrystals are formed at the SiO_2_/Ge/Ag interface, and the in-plane grain size decreases with increasing vertical distance from the silica substrate.

Decreasing size of Ag grains with growing distance from the substrate explains why the segregation of germanium in the silver structure occurs at an increasing rate. Ge atoms present in the boundaries of smaller nanocrystals have more neighboring crystals, what lowers the free enthalpy of such atoms. The opposite process, i.e., migration of Ge atoms from boundaries of smaller grains to boundaries of larger grains costs energy and thus should be possible only due to diffusion in a time scale longer than that of our measurements. After two months, the density slope of the silver film is inverted (Figure 3b) – during the segregation process, the Ge atoms fill the grain boundary voids, thus increasing the effective density (Figure 2, right). It is also worth noting, that in the case of modeling results of XRR measurements performed 3 days after the deposition (Figure 3a,c,e,g), it was necessary to consider a separate Ge interlayer between SiO_2_ substrate and silver, while in the case of measurements performed after 2 months (Figure 3b,d,f) it was not.

Calculated density profiles of Ag/Ge structures (Figure 3, right column) give information on dynamics of the segregation process. Annealing the samples at 100 °C results in the increase of the average grain size almost up to the value of the non wetted film (see Table 1). Annealing reduces the decrease of the silver film density with increasing distance from the SiO_2_/Ag interface, slowing down the segregation process. However, annealing at only 100 °C does not cancel the density slope completely, still allowing the segregation to occur, and that is the reason why after two months the annealed films exhibit a very similar segregation induced band in the Im(ε) permittivity spectrum as the non-annealed film [4]. Annealing the film at 300 °C results in the increase of the average grain size exactly up to the value of the non wetted film and completely cancels the density slope, which hinders the segregation process even more than in the case of annealing at 100 °C, but probably enhances diffusion. All samples have a similar lattice constant, though in the case of wetted films it is slightly higher than for the non wetted film. Annealing the wetted samples restores the lattice constant to the original value of 4.084 Å (see Table 1). It is also worth noting that oscillations in the XRR spectra in the case of measurements performed 2 months after the deposition process decay much faster than in the case of measurements performed 3 days after deposition, which indicates that with time, the surface roughness of the silver films has increased. This is likely because of the slight movement of the silver grains due to the migration of the Ge atoms to the subsurface grain boundaries.

**Figure 5:**
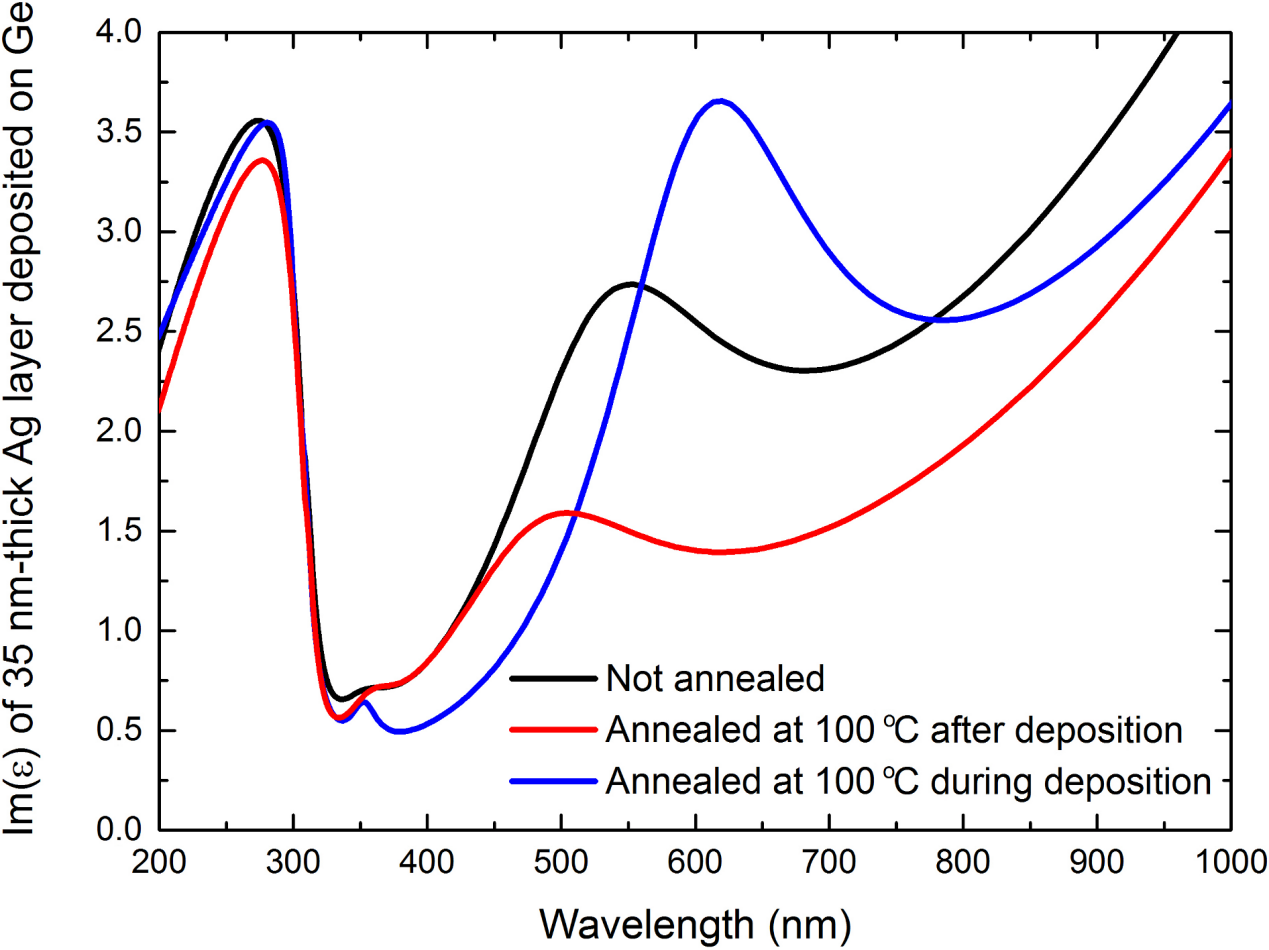
Imaginary part of permittivity for 35 nm thick Ag films deposited on 2 nm thick Ge wetting layer and capped with 3 nm thick LiF overlayer, measured using spectroscopic ellipsometry 10 days after the deposition process. The black and red curves have been reproduced from [4].

The XRR data for the samples annealed at 100 °C during deposition and measured 3 days after evaporation (Figure 3c) as well as annealed at 100 °C after deposition and measured 3 days afterwards (Figure 3e) are quite similar, and thus we assume that the crystalline structure is also similar. However, the dispersion relation of these films is different. Figure 5 shows the imaginary part of permittivity of 35 nm thick silver layers deposited on 2 nm thick Ge and capped with 3 nm thick LiF films, measured 10 days after deposition. It turns out, that even though in both cases the silver density slope is less slanted (Figure 3c,e) with respect to the non wetted film (Figure 3a), the segregation-induced peak decreased for the sample annealed after the deposition process (Figure 5, red line), but increased for the sample annealed during the deposition process (Figure 5, blue line). We believe that this is caused by the fact that deposition of silver on a hot substrate results in weaker wetting. Thus, even though germanium was utilized, silver formed islands during the deposition process – a transition from Frank–van der Merve to Volmer–Weber growth mode. This resulted in increasing the rate of the segregation process, since migration of Ge atoms on Ag grain surface is “easier” than migration through the grain boundaries, hence the increase in the segregation-induced band in the permittivity spectrum.

### Time evolution of Ge wetted Ag films

**Figure 6:**
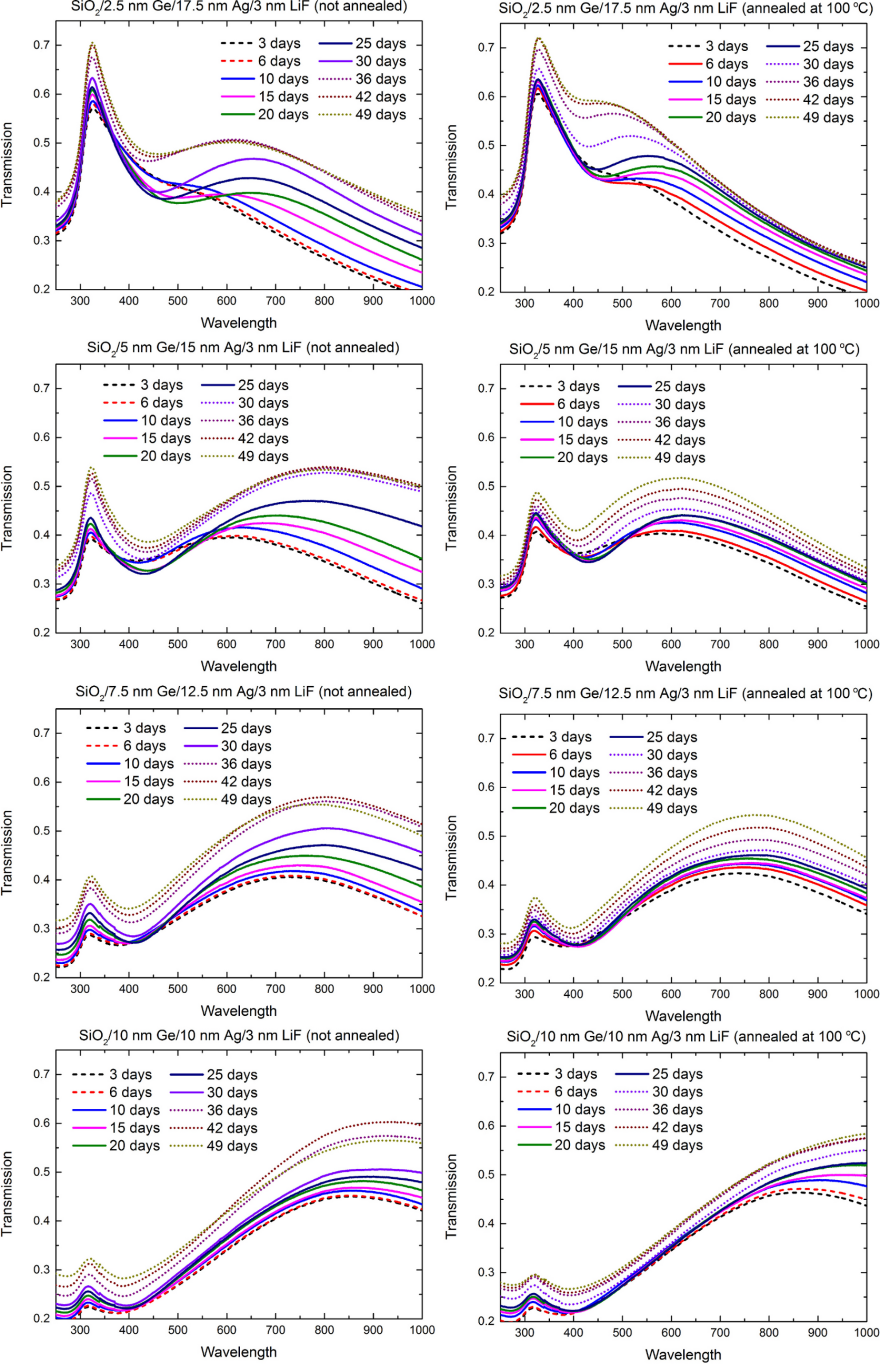
Transmission spectra of non-annealed (left column) and annealed (right column) Ag layers deposited on Ge wetting films, with constant sum of Ge and Ag thicknesses and varying Ge-to-Ag ratio. The Ge-to-Ag ratio changes from 1:7 (top row) to 1:1 (bottom row). The slight kinks at 340 and 370 nm are a result of changing the diffraction grating by the spectrometer.

We investigated the segregation rate of the Ge atoms within the Ag films structure as a function of the Ge-to-Ag thickness ratio. To this end, we have deposited silver on germanium with a fixed total thickness of both films equal to 20 nm, but with changing Ge-to-Ag ratio from 1:7 to 1:1, and recorded the transmission spectra of those samples over a period of 7 weeks. Time evolution of these transmission spectra is shown in Figure 6. In each case, three distinct stages were observed. At the stable stage (indicated by the dashed lines), which lasted up to 6 days after deposition, the transmission spectra did not change much. In the second period, which lasted from the 7th up to the 30th day (solid lines), the shape of the transmission curve underwent major changes – transmission decreased in the near-UV and short visible wavelengths but increased in the red and IR. For the sample SiO_2_/2.5 nm Ge/17.5 nm Ag/3 nm LiF, the transmission drops within the 375–550 nm wavelength range, with a minima at about 450 nm, but increases within the 550–1000 nm range with a maxima at about 650 nm. Increasing the proportion of germanium from 1:7 to 1:1 results in a blueshift of the minima and a redshift of the maxima in the transmission spectra. In the third stage (dotted lines), lasting since the 30th day, the transmission in the whole investigated spectrum increased.

**Figure 7:**
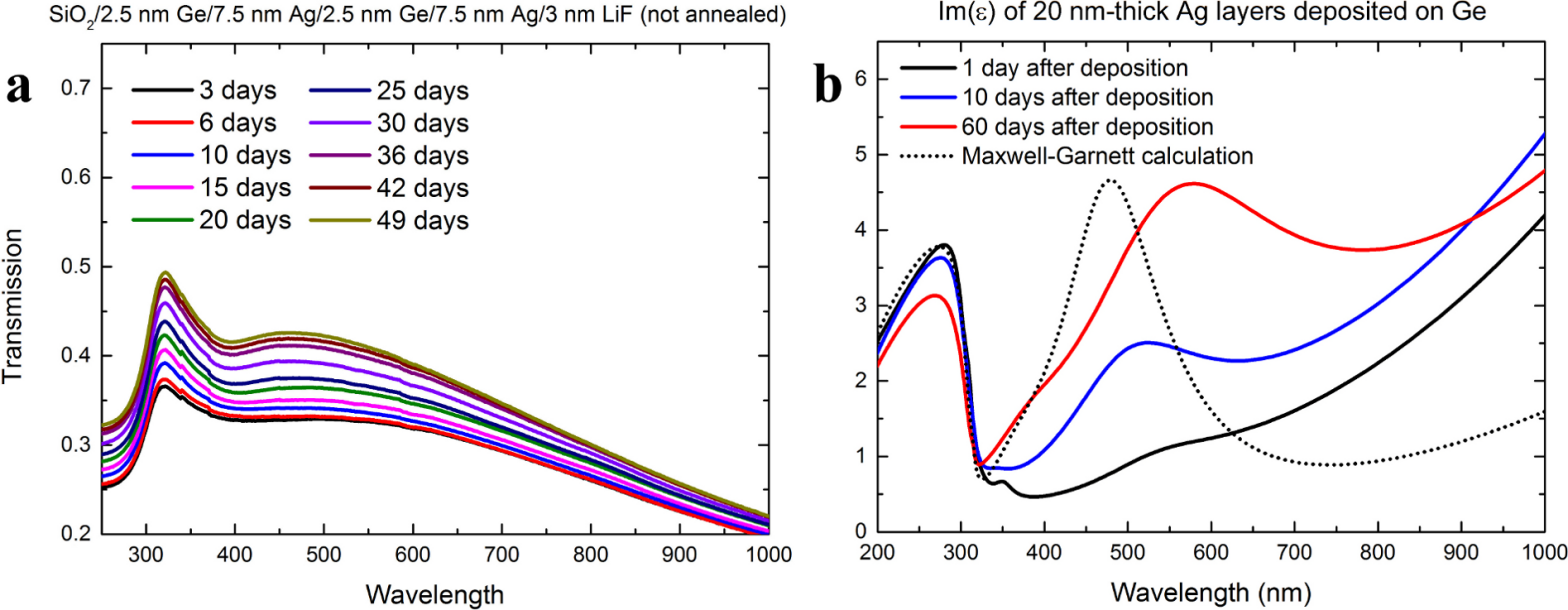
(a) Transmission spectra of Ge/Ag multilayer structure with total thickness of 20 nm. (b) Imaginary part of permittivity for 20 nm thick silver film deposited on 2 nm thick Ge wetting layer, measured using spectroscopic ellipsometry at 1, 10 and 60 days after deposition (solid lines), as well as Maxwell–Garnett calculation of the imaginary part of permittivity of Ge–Ag mixture with Ge-to-Ag ratio of 2:20 (dotted curve). Blue and red curves on the right figure have been reproduced from [4].

There is no reason for the segregation process not to begin at the time of deposition. This is confirmed by ellipsometric measurements – in the imaginary part of permittivity of a sample measured one day after the deposition (Figure 7b, black line), the segregation-induced band already exists, though it is very small. However, even if the segregation process is initiated at the time of deposition, the migration of Ge atoms through the grain boundaries of the large silver grains at the SiO_2_/Ge/Ag interface is probably slow. Due to horizontally elongated grains, the number of grain boundaries is small, and so, one Ge atom at the boundary essentially may prevent other atoms from segregating. Hence, the seemingly stable stage in the first days of the samples lifetime. The further the segregation process progresses, the faster it gets due to greater number of silver grain boundaries accessible to Ge atoms. Therefore, at a certain time, the influence of the segregation on the optical transmission spectra becomes noticeable and after that, the changes occur at a higher rate, which is what we have observed. After a certain amount of time, all of the Ge atoms segregate into the silver layer structure, and therefore, no further changes in the shape of the transmission curve should appear. The transmission results show that this segregation end point occurs at times not shorter than 25–30 days. This is confirmed by the Maxwell–Garnett calculation of effective permittivity of Ge inclusions embedded in silver with a Ge-to-Ag volume ratio of 1:10, which corresponds to 20 nm thick Ag film deposited on 2 nm thick Ge wetting layer. For these calculations, the permittivity of silver matrix was assumed as the permittivity of 20 nm thick silver layer deposited on glass [4] and the permittivity of Ge inclusions was accepted as the permittivity of 1 nm thick Ge layer measured in work [22]. The existence of a segregation-induced band is well predicted by the Maxwell–Garnet formula, with the exception, that the theoretical band is blueshifted by 100 nm with respect to the measurement. However, the value of the maximum of this band is almost identical in the case of calculation and the measurement performed 60 days after the deposition process, in which case we believe that the segregation has completed. For samples measured 1 or even 10 days after deposition (where segregation was still ongoing), the values of the maxima are much smaller than the value predicted by the Maxwell–Garnet formula. This is why we believe that the Maxwell–Garnett equation can be used to estimate the maximum value of the segregation-induced band for fully segregated samples. That said, it should be mentioned, that the Maxwell–Garnett approximation does not account for lowered contribution from the interband transitions (at wavelengths shorter than 300 nm) or for changes in the Drude term (increased imaginary part of permittivity at long wavelengths). In a long time scale, when segregation is completed, a competing process of grain boundary diffusion can still modify the distribution of Ge atoms within the silver layer, influencing the density slope and optical parameters [28].

For all non-annealed samples, regardless of the Ge-to-Ag ratio, the changes in transmission from one stage to another (from the stable period to the segregation-dominated one or from the segregation-dominated to the roughening period) are abrupt, precisely because of the increasing rate of the segregation process. For samples annealed at 100 °C, however, this transition is smoother. This is most likely due to the fact, that annealing decreases the slope of density profile of the silver layer, hindering the segregation process, but at the same time, it increases the roughness of the silver [4], which accelerates oxidation and sulfation. Even so, the transitions between stages can be easily spotted for samples with the thickness of Ge wetting film equal to 5 nm or less. If the thickness of the Ge layer is at least 7.5 nm (and the Ge-to-Ag ratio is 3:5 or greater), the evolution of transmission curves is very subtle and the transitions between the stable stage, segregation stage and roughening stage are hard to spot. This suggests that if a silver layer of thickness less than 15 nm is deposited on a germanium layer with a comparable thickness, such a system no longer exhibits segregation, but rather homogeneous mixing. This would also explain why for thick Ge layers, the changes in the transmission spectra during the segregation stage are much smaller than in the case of thinner Ge films.

Finally, to verify this, we have evaporated multilayer sample SiO_2_/2.5 nm Ge/7.5 nm Ag/2.5 nm Ge/7.5 nm Ag/3 nm LiF. This way, the Ge-to-Ag thickness ratio is as low as 1:3 (just as in the sample where 15 nm of Ag was deposited on 5 nm of Ge), but there should be intense mixing of the two materials. The transmission spectra are presented in Figure 7a. Unlike the transmission of the 5 nm Ge/15 nm Ag sample, which has the same Ge-to-Ag thickness ratio and exhibited pronounce changes during the segregation stage, the transmission of this sample only increases, most likely due to roughening. The changes resulting from segregation, such as lowered transmission in the near-UV and blue vs enhanced transition in red and IR, are almost non-noticeable. Therefore, we conclude that for Ag films of thickness lower than 15 nm, depositing them on Ge results in intense homogeneous elemental mixing rather than segregation.

### Raman spectra of Ge segregated in Ag

**Figure 8:**
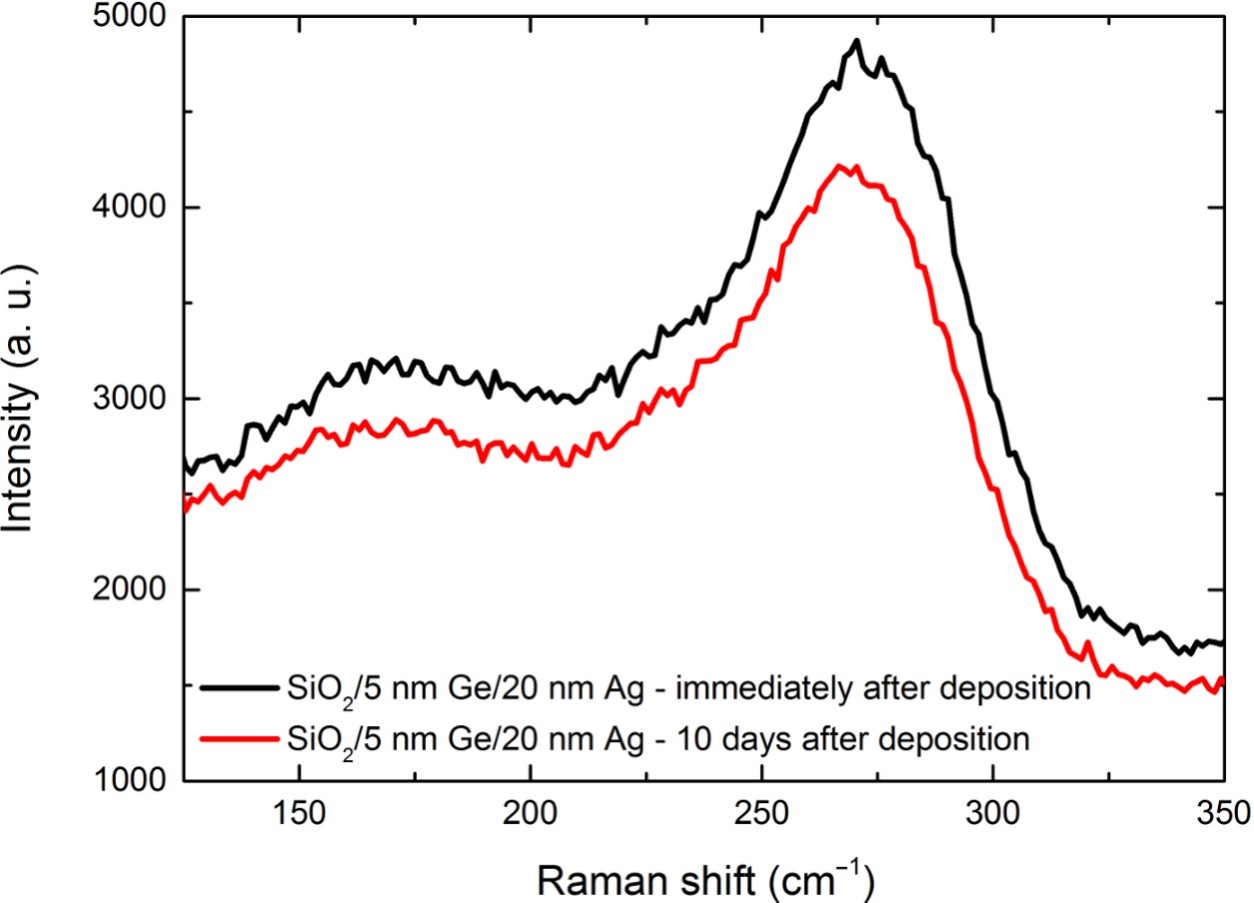
Raman spectra of 20 nm thick Ag layers deposited on 5 nm thick Ge wetting films.

To understand the nature of the segregation process we have fabricated 20 nm thick Ag layer wetted with 5 nm thick Ge film and performed Raman scattering measurements immediately after the deposition process as well as 10 days later. The results are presented in Figure 8. There are two characteristic bands in the Raman spectra. The band centered at 270 cm^−1^ most likely originates from the confined optical phonon in ultra-small germanium nanoclusters, while the band at around 160 nm is a combination of bands originating from transverse acoustic, longitudinal acoustic, longitudinal optical and transverse optical phonon modes in the amorphous Ge Raman spectrum [29–31]. This indicates a mixture of nanocrystalline and amorphous Ge. The bands are clearly visible even though the transparency of 20 nm thick Ag layer at these wavenumbers is extremely low (considering the fact that the laser wavelength is 532 nm). After 10 days of storing the sample in pressure below 10^−3^ Torr to reduce corrosion, the segregation is profound enough to noticeably influence the optical transmission, but the Raman spectrum barely changes. Due to germanium atoms distributed at the silver grain boundaries, which would no longer form Ge–Ge bonds (as it was in the case of a layer of Ge), and thus no longer conduct phonons, the peaks at 150–300 cm^−1^ should decrease significantly, yet the observed decrease barely reaches 13% at 269 cm^−1^. This suggests that segregating Ge atoms form clusters of a few atoms in which the Ge–Ge bonds are still present, although in a lesser number. This should still lower the Raman response more significantly than just the 13%. However, as it has been stated earlier, the segregation-induced band in the permittivity spectrum is a plasmonic one [4], so the signal from Ge clusters in the voids between silver grains is most likely enhanced by localized plasmons excited on the silver grains, in a similar way as SERS or TERS, hence only the slight drop in the intensity of the Raman response.

## Conclusion

Silver nanolayers deposited on ultrathin Ge wetting films exhibit gradient growth, with large and flat silver nanocrystals at the SiO_2_/Ge/Ag interfaces and decreasing grain size with increasing distance from the substrate. Such gradient growth is the most likely reason for the high-rate segregation of Ge atoms into the silver structure. The Raman spectra show that segregating germanium results in the formation of clusters of a few atoms, where Ge–Ge bonds are preserved. Normal transmission and ellipsometric measurements were used to investigate the kinetics of the segregation process. The first week after the deposition is a quasi-stable period, at which the evolution of dispersion is very weak. In the second stage, which lasts from the 7th up to the 30th day, the dispersion is strongly influenced by the segregation process, which typically completes within this period. After a month, aging effects appear – the samples start to roughen and corrode, which results in increased transmission. By applying temperature treatment to the sample at various stages of its evolution, the density slope of the silver layer (and thus the segregation kinetics) can be manipulated. Annealing the samples at temperatures close to the melting point (300 °C for 35 nm thick Ag layer) results in a uniform density of the silver layer, which ultimately terminates the segregation process.

## Experimental

All materials were deposited from fabmate or tungsten crucibles, using a PVD75 Lesker e-beam evaporator, on fused silica substrates with RMS roughness equal to 0.3 nm. The purity of the evaporation materials was 4N for silver, 5N for germanium and 3N for LiF. Before evaporation, substrates were cleaned with an argon flow at 2 bar pressure. Ge was evaporated at an average deposition rate of 0.5 Å/s. Silver was evaporated at an average deposition rate of 2 Å/s. LiF was evaporated at an average deposition rate of 1 Å/s to form a 3 nm thick capping layer. The deposition rate and total film thickness were monitored by two quartz weights inside the deposition chamber. Then, film thicknesses were verified by a Dektak 6M stylus profiler. The pressure in the vacuum chamber was kept below 5 × 10^−5^ Torr during the whole deposition process. The crucible-substrate distance was 40 cm.

AFM measurements were conducted on the day of the deposition of the samples using the Bruker Dimension Icon microscope working in the scan-assist mode at the solid–air interface. Cantilevers with spring constant *k* = 0.4 Nm^−1^ were used, the resonant frequency was in the range of 70–80 kHz. A typical image scan frequency was 1 Hz with a resolution of 512 × 512 px.

The X-ray reflectometry measurements were performed using a Bruker Discover D8 X-ray diffractometer working with Cu Kα line source of wavelength 0.154 nm, the diffraction signal were recorded with point scintillation detector. The monochromatic parallel beam was formed by a crossed parabolic Goebel mirrors. The data analysis was based on finding the proper electron density profile for which with XRR generated data matched the experimental one. Data fitting was performed using the Leptos 4.02 software package provided by Bruker. The electron density was simulated by ‘box’ type function. The thickness of the Ge wetting film was a fittable parameter (but the density of this film was fixed). The optical thickness of the Ag layer was fitted for the sample without the Ge wetting film, and then fixed for all other samples, while its density was left as a fittable parameter for all samples. The thickness and density of the LiF protective film were fitted for the sample without Ge wetting film, and then fixed for all other samples. The Ge/Ag and Ag/LiF interface roughness were left as fittable parameters. The roughness of the Ag/LiF interface estimated from the XRR models for samples measured 3 days after the deposition deviates no more than 0.3 nm from the values derived from the AFM scans. The only exception to that is the sample annealed at 300 °C, for which the general surface structure is similar to the structure of samples annealed at 100 °C, which results in similar roughness, but within every scan area of 3 × 3 µm^2^ there are a couple of very large grains (see Figure 2c), which taken into account the increase of the surface roughness of that sample to over 4 nm, which is similar to the value extracted from the XRR model. More information about XRR modeling can be found in [32] and references therein.

The wide-angle X-ray diffraction (XRD) measurements were performed in transmission mode using a Bruker Discover D8 GADDS system. The system works with Cu Kα X-ray source. The X-ray patterns are recorded with 2D Vantec 2000 detector. For precise diffraction angle measurements, also a Bruker Discover D8 system was used, but the measurements were performed in reflection geometry in θ − 2θ scans. The X-ray signals were recorded with 1D Vantec-1 detector. The width and position of the signals were analyzed with TOPAS software. The average size of silver grain was then calculated by fitting the Gaussian profile to the dominant diffraction peak at 38.2°, which corresponds to (111) orientation of grains with respect to the c axis. Then the full width at half maximum (FWHM) parameter of the fitted Gaussian profile was used in the Debye–Scherer formula:

[1]
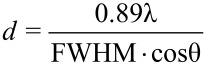


where *d* is the average grain size, λ is the incident wavelength (in this case 0.154 nm) and θ is the Bragg diffraction angle. The lattice constant was then derived from the position of the fitted Gaussian profile.

Ellipsometric azimuths Ψ and Δ of fabricated samples were measured in the UV–vis–MIR spectral range (0.06–6.5 eV) for three angles of incidence (65°, 70° and 75°) by the use of two instruments: V-VASE (J.A.Woollam Co., Inc.) in the UV–vis–NIR and Sendira (Sentech GmbH) in the MIR. The complex dielectric functions of effective Ag layers with segregated Ge atoms were extracted using a layered model of the samples. The permittivity’s were then interpreted in terms of the Lorentz, Drude–Lorentz and modified Lorentz [33] oscillator models. In case of theoretically calculated permittivity curves for silver layers with Ge atoms segregated in them, the following Maxwell–Garnett equation was used

[2]
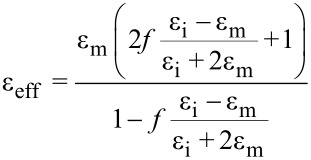


where ε_eff_ is the effective permittivity of the system, ε_m_ the permittivity of the medium (in this case the Ag layer) ε_i_ the permittivity of the inclusion (in this case the Ge atoms) and *f* is the inclusion fill factor.

Normal transmission spectra were recorded using METASH UV-6000 spectrophotometer. The transmission curves were not normalized to a blank substrate as this would introduce additional errors originating from different reflection at air/glass and silver/glass interfaces.

Raman spectra were recorded using Renishaw invia RE04 spectrometer equipped with an excitation source of Nd:YAG laser operating at 532 nm wavelength. Laser spot of 1.5 μm diameter was obtained by applying an objective with a 50× magnification. The laser power was kept at 1 mW to avoid damage or heating.
